# Acute toxicity and sex-dependent lethality of isotonitazene in fischer 344 rats

**DOI:** 10.3389/ftox.2026.1818684

**Published:** 2026-04-01

**Authors:** Yesenia Lopez Hernandez, Horatiu Vinerean, Saurabh Aggarwal

**Affiliations:** 1 Department of Cellular and Molecular Medicine, Herbert Wertheim College of Medicine, Florida International University, Miami, FL, United States; 2 The Office of Laboratory Animal Research, Florida International University, Miami, FL, United States

**Keywords:** acute toxicity, isotonitazene, LD50, nitazenes, respiratory depression, sex differences

## Abstract

Isotonitazene is a highly potent benzimidazole-derived synthetic opioid increasingly implicated in fatal intoxications. Despite growing forensic detection, controlled *in vivo* toxicity data remain limited. We determined the median lethal dose (LD_50_) of isotonitazene in adult male and female Fischer 344 rats using the Dixon up-and-down procedure following single intraperitoneal administration. Mortality and clinical signs were monitored for 14 days. The estimated LD_50_ was 0.08 mg/kg (95% CI 0.02996–0.182 mg/kg) in males and 0.25 mg/kg (95% CI 0.04841–0.768 mg/kg) in females, indicating greater male sensitivity. Rapid respiratory depression and neuromuscular rigidity occurred within minutes of dosing and preceded lethality. The narrow separation between survival and death demonstrates a steep dose–response relationship and limited safety margin. These findings provide quantitative data for toxicological risk assessment of nitazene opioids and underscore the importance of sex as a biological variable in opioid toxicity studies.

## Introduction

The global opioid crisis has evolved through multiple phases characterized by the emergence of increasingly potent synthetic opioids. While early phases were dominated by prescription opioids and heroin, recent years have been marked by the proliferation of highly potent synthetic opioids in illicit drug markets, creating significant challenges for public health, clinical management, and forensic toxicology. Although fentanyl and its analogues have been central contributors to overdose mortality, a growing number of non-fentanyl synthetic opioids (NFSOs) have recently emerged, many of which belong to the benzimidazole-derived opioid class known as nitazenes (Shover et al., 2021; Tettey et al., 2018; Jadhav and Fasinu, 2024; DEA, 2025).

Nitazenes are synthetic opioids based on the 2-benzylbenzimidazole scaffold, originally developed in the 1950 s during pharmaceutical efforts to identify potent analgesics. Early pharmacological investigations demonstrated that several compounds within this class possess exceptionally high potency at the µ-opioid receptor (MOR), often exceeding that of morphine and approaching or surpassing the activity of fentanyl. Despite their potent analgesic properties, most nitazene derivatives were never developed for therapeutic use due to concerns regarding severe respiratory depression, narrow therapeutic (analgesia) margins, and high abuse potential. After remaining largely absent from the pharmaceutical landscape for decades, these compounds have re-emerged in recent years as new psychoactive substances (NPS) within illicit opioid markets.

Among these compounds, isotonitazene has become one of the most frequently detected nitazene opioids in forensic toxicology investigations (Pereira et al., 2025). First identified in illicit drug supplies around 2019, isotonitazene was initially misidentified as etonitazene before subsequent structural characterization confirmed its identity (Blanckaert et al., 2020; Zagorski et al., 2020). Since its emergence, isotonitazene has been increasingly reported in seized drug materials, clinical toxicology cases, and post-mortem investigations across North America and Europe (Krotulski et al., 2020; Mueller et al., 2021). Epidemiological studies and forensic analyses have linked isotonitazene to numerous fatal intoxications, often in combination with other substances such as benzodiazepines, stimulants, or other opioids (Krotulski et al., 2020; Mueller et al., 2021; Shover et al., 2021). Recent surveillance reports have highlighted the growing presence of nitazenes within illicit drug markets and their potential contribution to opioid-related mortality.

Pharmacologically, nitazenes act as high-efficacy agonists at the µ-opioid receptor, the primary receptor responsible for opioid-induced analgesia and respiratory depression (Kozell et al., 2024; Hataoka et al., 2025). *In vitro* receptor binding and functional assays have demonstrated that several nitazene derivatives possess nanomolar potency and high intrinsic efficacy, which may explain their capacity to produce profound respiratory depression at very low doses (Kozell et al., 2024; Malcolm et al., 2023; Rombola et al., 2025). These properties raise significant toxicological concerns, particularly when these compounds are distributed illicitly without dose control and may be misrepresented as other opioids such as heroin or fentanyl. Despite growing forensic and epidemiological evidence, controlled experimental data describing the *in vivo* toxicological properties of nitazenes remain limited. Much of the current knowledge regarding these compounds derives from case reports, toxicological surveillance, or *in vitro* pharmacological studies. In particular, experimentally derived dose-response relationships and acute lethality data for many nitazene analogues remain poorly characterized. Establishing quantitative measures of toxicity, such as the median lethal dose (LD_50_), is an important component of toxicological evaluation because it provides insight into acute toxicity profiles and dose-response relationships under controlled conditions.

Another factor that may influence opioid toxicity is biological sex. Sex differences have been reported for several opioids with respect to analgesic responses, respiratory depression, and pharmacokinetic properties (DeVito et al., 2024; Khandaker et al., 2025; Towers et al., 2024; Walter et al., 2022; Wightman et al., 2021). These differences may reflect variation in µ-opioid receptor expression, hormonal modulation of respiratory control networks, and sex-dependent drug metabolism. Emerging evidence also suggests that nitazene opioids undergo complex metabolic pathways involving cytochrome P450 enzymes, including CYP2D6 and CYP3A4, which may contribute to variability in systemic exposure and toxicity (Jadhav and Fasinu, 2024). However, the extent to which such factors influence the acute toxicity of nitazene compounds remains largely unknown. Among the various nitazene derivatives currently detected in illicit drug markets, isotonitazene has attracted particular attention due to its frequent detection in toxicological casework and its suspected involvement in fatal overdoses. Despite this growing concern, controlled *in vivo* studies examining the acute toxicity of isotonitazene remains scarce.

Accordingly, the present study aimed to determine the acute median lethal dose (LD_50_) of isotonitazene in male and female Fischer 344 rats following intraperitoneal administration. LD_50_ values were estimated using the Dixon up-and-down procedure, a sequential dosing approach that enables efficient estimation of lethal dose parameters while minimizing animal use. By characterizing the acute toxicity of isotonitazene and exploring potential sex-dependent differences in lethality, this study provides experimental data relevant to the toxicological assessment of emerging nitazene opioids and contributes to a better understanding of their potential risks in both clinical and forensic contexts.

## Materials and methods

### Animals

Adult male and female Fischer 344 rats were obtained from a commercial breeder and acclimated for at least 1 week before experimentation. At the beginning of the study, animals were 14–16 weeks of age. Male rats weighed 300–350 g, and female rats weighed 180–220 g. Animals were housed under controlled environmental conditions (22 °C ± 2 °C; 12-h light/dark cycle) with *ad libitum* access to standard laboratory chow and water. Rats were group-housed in standard polycarbonate cages with environmental enrichment. All animals were drug-naïve and fully conscious at the time of dosing. After drug administration, animals were returned to their home cages and observed according to the monitoring protocol. All procedures were approved by the Institutional Animal Care and Use Committee (IACUC) of Florida International University (Protocol No. 24–069) and conducted in accordance with the ARRIVE guidelines.

### Test substance

Isotonitazene (Cayman Chemical, Catalog No. 27255; purity >98%) was prepared immediately prior to administration. The compound was dissolved in 20% dimethyl sulfoxide (DMSO) in saline to ensure adequate solubility and homogeneous dosing. Intraperitoneal injections were administered in volumes adjusted according to body weight.

### Study design and LD_50_ determination

Acute toxicity was assessed following single intraperitoneal (IP) administration of isotonitazene. Median LD_50_ values were estimated using the Dixon up-and-down procedure, a sequential dosing method commonly used in toxicological studies to estimate LD_50_ while minimizing the number of animals required (Dixon, 1980). In this method, the dose administered to each subsequent animal is determined based on the survival outcome of the previous animal. If the animal survives, the dose for the next subject is increased; if mortality occurs, the dose is decreased. Sequential dosing continues until sufficient dose reversals occur to permit LD_50_ estimation. Dose levels evaluated in the present study included: 0.025 mg/kg, 0.08 mg/kg, 0.25 mg/kg, and 0.5 mg/kg. The study was initially conducted on male rats using a 0.5 mg/kg dose. This dose was selected based upon the known median lethal dose for Etonitazene (a close analog of Isotonitazene) in rabbits (Gross and Turrian, 1957). Subsequently, for female rats, 0.25 mg/kg dose was used as the initial dose as 0.5 mg/kg caused 100% mortality in male rats.

Animals were continuously observed for the first hour following dosing to record acute clinical signs, including respiratory depression, neuromuscular rigidity, and behavioral abnormalities. Respiratory rate was assessed through direct observation of thoracic movements during defined observation intervals. Following the acute observation period (1 h), animals were monitored daily for 14 days for delayed mortality or clinical abnormalities. Observations included posture, locomotor activity, grooming behavior, and general physical condition. Approximate time-to-death was recorded for animals that did not survive.

### Statistical analysis

LD_50_ values and 95% confidence intervals were calculated using probit-based methods appropriate for UDP designs. Analyses were performed separately for males and females.

## Results

Fischer 344 rats were exposed to isotonitazene (IP) and observed continuously for 1 h (short term) and then subsequently daily for 14 days (long-term). Isotonitazene produced mortality within a narrow dose range (small difference between doses producing survival and mortality, suggesting a steep dose-response relationship) in both sexes. In males, survival occurred at 0.025 mg/kg, while mortality was observed at 0.25 and 0.5 mg/kg. Mixed outcomes at 0.08 mg/kg yielded an estimated LD_50_ of 0.08 mg/kg (95% CI. 0.02996–0.182 mg/kg) (Table 1). In females, survival occurred at 0.08 mg/kg, whereas mortality was observed at 0.5 mg/kg. Mixed outcomes at 0.25 mg/kg yielded an estimated LD_50_ of 0.25 mg/kg (95% CI 0.04841–0.768 mg/kg) (Table 1). Males exhibited approximately threefold greater sensitivity than females. Mortality typically occurred within approximately 10–30 min after dosing at lethal dose levels.

**TABLE 1:** LD50: Dixon up-and-down dosing sequences and survival outcomes following single-dose intraperitoneal administration of isotonitazene in adult Fischer 344 rats. Each row represents one animal and the administered dose level. Short-term (1-h post) and long-term (14-day post) outcomes are shown for each subject. X indicates mortality, and O indicates survival. Male rats received 0.025, 0.08, 0.25, and 0.5 mg/kg doses. While female rats received 0.08, 0.25, and 0.5 mg/kg doses.

Male rat ID	Dose (mg/kg)	Short-term result	Long-term result	Female rat ID	Dose (mg/kg)	Short-term result	Long-term result
M1	0.5	X	X	F1	0.25	X	X
M2	0.25	X	X	F2	0.08	O	O
M3	0.08	X	X	F3	0.25	X	X
M4	0.025	O	O	F4	0.08	O	O
M5	0.08	O	O	F5	0.25	O	O
M6	0.25	X	X	F6	0.5	X	X
M7	0.08	X	X	​	​	​	​
M8	0.025	O	O	​	​	​	​
M9	0.08	O	O	​	​	​	​
​	**LD50 = 0.08**	X = died	O = survived	​	**LD50 = 0.25**	X = died	O = survived

Bold values denote LD50 values.

Respiratory depression preceded mortality dose dependently. Male rats exhibited greater respiratory depression than females at comparable dose levels, supporting a sex-dependent difference in acute toxicodynamic response (Table 2). Although male animals exhibited a lower estimated LD_50_ and greater respiratory depression at comparable dose levels, the confidence intervals for LD_50_ estimates overlapped, indicating that additional studies are required to determine whether these observations represent true sex-dependent differences.

**TABLE 2:** RR: Dose-dependent respiratory rate (RR) changes in male and female Fischer 344 rats measured 10 min after intraperitoneal isotonitazene administration. Values represent observed average RR per minute and % decline from the baseline at each tested dose level.

Male rat dose (mg/kg)	Male RR/min	Male RR (% decline from the baseline)	Female rat dose (mg/kg)	Female RR/min	Female RR (% decline from the baseline)
0.5	20	82	0.5	35	68
0.25	36	67	0.25	56	49
0.08	53	51	0.08	110	0
0.025	110	0	​	​	​

**FIGURE 1:**
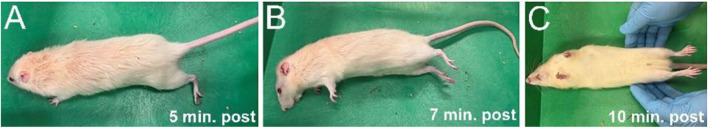
Sequential photographs of a rat obtained at 5, 7, and 10 min after intraperitoneal isotonitazene administration **(A–C)**, illustrating time-dependent changes in posture, activity level, and observable body rigidity.

At LD_50_ and higher doses, animals showed rapid-onset of neuromuscular signs that developed within minutes of dosing and progressed in a consistent sequence (Figure 1). Rats exhibited trunk, neck, and jaw rigidity within ∼5 min, followed by upper-limb flexion and hand clenching by ∼7 min, and lower-limb extension by ∼10 min, indicating a rapid-onset, severe motor syndrome.

## Discussion

The present study provides controlled experimental data describing the acute toxicity of isotonitazene in male and female Fischer 344 rats. Using the Dixon up-and-down procedure, we estimated median lethal dose (LD_50_) values of 0.08 mg/kg in males and 0.25 mg/kg in females following intraperitoneal administration. Mortality occurred rapidly following dosing and was preceded by pronounced respiratory depression and neuromuscular rigidity. These findings demonstrate that isotonitazene produces severe acute opioid toxicity at sub-milligram per kilogram doses in this rodent model.

### Comparison with other opioids

When interpreted within the broader context of opioid toxicology, the LD_50_ values observed in this study indicate that isotonitazene possesses marked acute toxicity in rodents. However, direct comparisons between compounds across different studies should be interpreted cautiously due to differences in experimental conditions, including species, strain, sex, route of administration, and methodological approaches used to estimate LD_50_ values. Previous reports have described LD_50_ for fentanyl (subcutaneous) in mice to be 135 mg/kg (Newman et al., 2024), whereas morphine typically exhibits substantially higher LD_50_ values in mice (400 mg/kg) (Grant et al., 1994) depending on strain and experimental conditions. Although the present study did not directly compare these opioids under identical conditions, the sub-milligram LD_50_ values observed for isotonitazene suggest very high acute toxicity consistent with the pharmacological properties of nitazene opioids. Historically, several nitazene derivatives, including etonitazene and related compounds, have demonstrated potent opioid activity in animal models (May et al., 1999). However, many early pharmacological studies employed different experimental designs, species, and routes of administration, which complicates direct quantitative comparison (Gross and Turrian, 1957). Consequently, the LD_50_ values reported here should primarily be interpreted as controlled estimates of isotonitazene lethality within the parameters of the present experimental model.

### Mechanisms of acute toxicity

The clinical signs observed in this study are consistent with severe opioid-induced respiratory depression, which represents the primary mechanism of fatal opioid intoxication. Animals receiving doses near or above the LD_50_ exhibited rapid decreases in respiratory rate accompanied by progressive neuromuscular rigidity. These signs developed within minutes of dosing, indicating rapid systemic absorption and central nervous system penetration following intraperitoneal administration. The observed truncal and cervical rigidity resembles the “wooden chest syndrome” described with high-potency opioids such as fentanyl (Pergolizzi et al., 2021; Wei et al., 2026). This phenomenon involves opioid-mediated activation of central motor pathways, producing increased muscle tone that can mechanically impair ventilation (Wei et al., 2026; Haouzi and Tubbs, 2022). In addition to direct suppression of brainstem respiratory centers through µ-opioid receptor activation, this rigidity may further exacerbate hypoxia by limiting thoracic expansion. Nitazene opioids are known to function as high-efficacy µ-opioid receptor agonists, and several *in vitro* studies have reported receptor activation profiles comparable to or exceeding those of fentanyl (Vandeputte et al., 2024). High intrinsic efficacy at the µ-opioid receptor may therefore contribute to the rapid and severe respiratory depression observed in the present study.

### Potential role of drug metabolism

Although pharmacokinetic measurements were not obtained in the present study, studies show that opioids such as fentanyl and morphine achieve peak plasma concentration within 4–90 min, depending on the route of administration (Wickham, 2017; Moss and Carlo, 2019; Lotsch et al., 2013; Lugo et al., 2005). In addition, emerging evidence suggests that nitazenes undergo complex hepatic metabolism involving multiple cytochrome P450 enzymes, including CYP3A4 and CYP2D6 (Jadhav and Fasinu, 2024). Recent *in vitro* investigations using human hepatocytes have demonstrated that benzimidazole opioids may undergo oxidative metabolism and subsequent conjugation reactions that influence systemic exposure and metabolite formation (Jadhav and Fasinu, 2025). Genetic polymorphisms in enzymes such as CYP2D6 can produce substantial interindividual differences in drug metabolism in humans (Chiba et al., 2012). While the relevance of these pathways to isotonitazene toxicity remains incompletely characterized, variability in metabolic clearance may contribute to differences in systemic exposure and overdose risk. Future studies incorporating pharmacokinetic profiling will be important for clarifying the relationship between systemic drug concentrations and toxicological outcomes.

### Sex-dependent differences in toxicity

A difference in estimated LD_50_ values was observed between male and female rats, with males demonstrating a lower LD_50_ than females in the present study. Male animals also exhibited greater respiratory depression at comparable dose levels. These observations suggest a potential sex-dependent difference in sensitivity to isotonitazene. However, it is important to interpret this finding cautiously. The Dixon up-and-down method uses relatively small sample sizes, and the confidence intervals for the LD_50_ estimates partially overlapped, indicating that additional studies with larger cohorts will be required to confirm whether this difference reflects a true biological effect. Sex differences in opioid pharmacology have been reported previously and may arise from multiple factors, including variation in µ-opioid receptor expression, hormonal modulation of respiratory control circuits, and sex-dependent drug metabolism (Bernal et al., 2007; DeVito et al., 2024; Ethridge and Smith, 2023; Kest et al., 1999; Khandaker et al., 2025; Lopes et al., 2021; Loyd et al., 2008; Sarton et al., 1999; Vevelstad et al., 2026; Wightman et al., 2021). However, previous studies have also demonstrated that the direction and magnitude of sex differences in opioid sensitivity can vary depending on species and strain. As a result, the present observations should be interpreted as indicating a possible trend rather than definitive evidence of sex-specific toxicity.

### Toxicological and public health implications

The extremely low LD_50_ values observed in this study highlight the potential toxicological risk posed by nitazene opioids. In illicit drug markets, these substances may be distributed without dosing control and may be misrepresented as other opioids, increasing the likelihood of unintentional overdose (Pucci et al., 2024). The rapid onset of respiratory depression observed in this study also suggests that intoxication may progress quickly following exposure, potentially limiting the window for medical intervention. The increasing detection of nitazenes in forensic casework and toxicological investigations underscores the need for improved toxicological characterization of these compounds, including pharmacokinetic studies, receptor pharmacology, and evaluation of antidote responsiveness (Kashindye et al., 2026; Krotulski et al., 2020; Pereira et al., 2025; Tettey and Crean, 2015; Vevelstad et al., 2026).

## Limitations

Several limitations should be considered when interpreting the findings of this study. First, the Dixon up-and-down procedure is designed to estimate LD_50_ values using relatively small numbers of animals, which may limit statistical power for detecting subtle differences between groups. Second, the study was conducted in a single rat strain using a single route of administration, which may limit extrapolation to other experimental models or routes relevant to human exposure. Additionally, pharmacokinetic measurements were not obtained, and therefore the relationship between systemic drug concentrations and toxicological effects could not be assessed. Future investigations incorporating pharmacokinetic analysis and larger experimental cohorts will be important for further characterizing the toxicity profile of isotonitazene.

## Conclusion

In summary, isotonitazene demonstrated pronounced acute toxicity in Fischer 344 rats, with LD_50_ values in the sub-milligram per kilogram range following intraperitoneal administration. Severe respiratory depression and neuromuscular rigidity occurred rapidly after dosing and preceded mortality. A lower estimated LD_50_ was observed in male rats compared with females, suggesting a potential sex-dependent difference in sensitivity, although additional studies are required to confirm this finding.

These results contribute to the limited experimental literature describing the toxicological properties of emerging nitazene opioids and highlight the need for continued pharmacological and toxicological investigation of this rapidly evolving class of opioids.

## Data Availability

The raw data supporting the conclusions of this article will be made available by the authors, without undue reservation.

## References

[B1] Bernal S. A. Morgan M. M. Craft R. M. (2007). PAG mu opioid receptor activation underlies sex differences in morphine antinociception. Behav. Brain Res. 177, 126–133. 10.1016/j.bbr.2006.10.028 17118467 PMC1868665

[B2] Blanckaert P. Cannaert A. Van Uytfanghe K. Hulpia F. Deconinck E. Van Calenbergh S. (2020). Report on a novel emerging class of highly potent benzimidazole NPS opioids: Chemical and *in vitro* functional characterization of isotonitazene. Drug Test. Anal. 12, 422–430. 10.1002/dta.2738 31743619

[B3] Chiba K. Kato M. Ito T. Suwa T. Sugiyama Y. (2012). Inter-individual variability of *in vivo* CYP2D6 activity in different genotypes. Drug Metab. Pharmacokinet. 27, 405–413. 10.2133/dmpk.dmpk-11-rg-078 22277677

[B4] DEA (2025). National drug threat assessment. Editor Administration D. E.

[B5] Devito E. E. Ameral V. Sofuoglu M. (2024). Sex differences in comorbid pain and opioid use disorder: a scoping review. Br. J. Clin. Pharmacol. 90, 3067–3083. 10.1111/bcp.16218 39168150 PMC11604518

[B6] Dixon W. J. (1980). Efficient analysis of experimental observations. Annu. Rev. Pharmacol. Toxicol. 20, 441–462. 10.1146/annurev.pa.20.040180.002301 7387124

[B7] Ethridge S. B. Smith M. A. (2023). Estradiol and Mu opioid-mediated reward: the role of estrogen receptors in opioid use. Addict. Neurosci. 9, 100139. 10.1016/j.addicn.2023.100139 38155959 PMC10753849

[B8] Grant G. J. Vermeulen K. Zakowski M. I. Stenner M. Turndorf H. Langerman L. (1994). Prolonged analgesia and decreased toxicity with liposomal morphine in a mouse model. Anesth. Analg. 79, 706–709. 10.1213/00000539-199410000-00015 7943779

[B9] Gross F. Turrian H. (1957). Benzimidazole derivatives with strong analgesic effects. Experientia 13, 401–403. 10.1007/BF02161117 13473818

[B10] Haouzi P. Tubbs N. (2022). Effects of fentanyl overdose-induced muscle rigidity and dexmedetomidine on respiratory mechanics and pulmonary gas exchange in sedated rats. J. Appl. Physiol. 132, 1407–1422. 10.1152/japplphysiol.00819.2021 35421320 PMC9190730

[B11] Hataoka K. Hojo M. Nomura S. Nakagawa Y. Kawai A. Nakamura M. (2025). Evaluation of rewarding effects of nitazene analogs: results from conditioned place preference tests and *in vivo* microdialysis experiments in mice. J. Toxicol. Sci. 50, 33–43. 10.2131/jts.50.33 39779230

[B12] Jadhav G. R. Fasinu P. S. (2024). Metabolic characterization of the new benzimidazole synthetic opioids - nitazenes. Front. Pharmacol. 15, 1434573. 10.3389/fphar.2024.1434573 39092223 PMC11291330

[B13] Jadhav G. R. Fasinu P. S. (2025). Biotransformation and kinetics of selected benzimidazole synthetic opioids in human hepatocytes. Front. Pharmacol. 16, 1656437. 10.3389/fphar.2025.1656437 41394146 PMC12695736

[B14] Kashindye R. C. Yadav D. Yadav R. (2026). Unraveling isotonitazene: insights into chemistry, pharmacology, and analytical techniques in forensic toxicology. J. Forensic Leg. Med. 118, 103069. 10.1016/j.jflm.2026.103069 41490351

[B15] Kest B. Wilson S. G. Mogil J. S. (1999). Sex differences in supraspinal morphine analgesia are dependent on genotype. J. Pharmacol. Exp. Ther. 289, 1370–1375. 10.1016/S0022-3565(24)38281-3 10336528

[B16] Khandaker R. Eyth A. Eikermann G. M. Carroll M. Rudolph M. Grimm A. M. (2025). Sex differences in opioid-related mortality trends in the United States, 1999-2020: by age, race/ethnicity, region, and opioid type. J. Womens Health (Larchmt) 34, 1451–1461. 10.1177/15409996251369454 40824729

[B17] Kozell L. B. Eshleman A. J. Wolfrum K. M. Swanson T. L. Bloom S. H. Benware S. (2024). Pharmacologic characterization of substituted nitazenes at mu, kappa, and Delta opioid receptors suggests high potential for toxicity. J. Pharmacol. Exp. Ther. 389, 219–228. 10.1124/jpet.123.002052 38453524 PMC11026150

[B18] Krotulski A. J. Papsun D. M. Kacinko S. L. Logan B. K. (2020). Isotonitazene quantitation and metabolite discovery in authentic forensic casework. J. Anal. Toxicol. 44, 521–530. 10.1093/jat/bkaa016 32091095

[B19] Lopes G. S. Bielinski S. Moyer A. M. Jacobson D. J. Wang L. Jiang R. (2021). Sex differences in type and occurrence of adverse reactions to opioid analgesics: a retrospective cohort study. BMJ Open 11, e044157. 10.1136/bmjopen-2020-044157 34193479 PMC8246359

[B20] Lotsch J. Walter C. Parnham M. J. Oertel B. G. Geisslinger G. (2013). Pharmacokinetics of non-intravenous formulations of fentanyl. Clin. Pharmacokinet. 52, 23–36. 10.1007/s40262-012-0016-7 23100195

[B21] Loyd D. R. Wang X. Murphy A. Z. (2008). Sex differences in micro-opioid receptor expression in the rat midbrain periaqueductal gray are essential for eliciting sex differences in morphine analgesia. J. Neurosci. 28, 14007–14017. 10.1523/JNEUROSCI.4123-08.2008 19109484 PMC2819468

[B22] Lugo R. A. Satterfield K. L. Kern S. E. (2005). Pharmacokinetics of methadone. J. Pain Palliat. Care Pharmacother. 19, 13–24. 10.1080/J354v19n04_05 16431829

[B23] Malcolm N. J. Palkovic B. Sprague D. J. Calkins M. M. Lanham J. K. Halberstadt A. L. (2023). Mu-opioid receptor selective superagonists produce prolonged respiratory depression. iScience 26, 107121. 10.1016/j.isci.2023.107121 37416459 PMC10320493

[B24] May T. Juilfs F. Wolffgramm J. (1999). Effects of etonitazene consumption and abstinence on the signal transmission of mu-opioid receptors in brain membranes of rats. Neurosci. Lett. 275, 109–112. 10.1016/s0304-3940(99)00741-7 10568511

[B25] Moss R. B. Carlo D. J. (2019). Higher doses of naloxone are needed in the synthetic opiod era. Subst. Abuse Treat. Prev. Policy 14, 6. 10.1186/s13011-019-0195-4 30777088 PMC6379922

[B26] Mueller F. Bogdal C. Pfeiffer B. Andrello L. Ceschi A. Thomas A. (2021). Isotonitazene: fatal intoxication in three cases involving this unreported novel psychoactive substance in Switzerland. Forensic Sci. Int. 320, 110686. 10.1016/j.forsciint.2021.110686 33497988

[B27] Newman M. Connery H. Kannan S. Gautam A. Hammamieh R. Chakraborty N. (2024). Fentanyl overdose causes prolonged cardiopulmonary dysregulation in Male SKH1 mice. Pharm. (Basel) 17, 941. 10.3390/ph17070941 39065791 PMC11279777

[B28] Pereira J. R. P. Quintas A. Neng N. R. (2025). Nitazenes: the emergence of a potent synthetic opioid threat. Molecules 30, 3890. 10.3390/molecules30193890 41097311 PMC12526229

[B29] Pergolizzi J. V. Jr. Webster L. R. Vortsman E. Ann Lequang J. Raffa R. B. (2021). Wooden chest syndrome: the atypical pharmacology of fentanyl overdose. J. Clin. Pharm. Ther. 46, 1505–1508. 10.1111/jcpt.13484 34240442

[B30] Pucci M. Singh Jutley G. Looms J. Ford L. (2024). N-desethyl isotonitazene detected in polydrug users admitted to hospital in Birmingham, United Kingdom. Clin. Toxicol. (Phila) 62, 19–25. 10.1080/15563650.2024.2309321 38353737

[B31] Rombola F. Bartoletti S. Bilel S. Hrelia P. Marti M. Lenzi M. (2025). *In vitro* cytotoxic and genotoxic evaluation of nitazenes, a potent class of new synthetic opioids. J. Xenobiot. 15, 203. 10.3390/jox15060203 41440750 PMC12733476

[B32] Sarton E. Teppema L. Dahan A. (1999). Sex differences in morphine-induced ventilatory depression reside within the peripheral chemoreflex loop. Anesthesiology 90, 1329–1338. 10.1097/00000542-199905000-00017 10319781

[B33] Shover C. L. Falasinnu T. O. Freedman R. B. Humphreys K. (2021). Emerging characteristics of isotonitazene-involved overdose deaths: a case-control study. J. Addict. Med. 15, 429–431. 10.1097/ADM.0000000000000775 33234804 PMC8141068

[B34] Tettey J. Crean C. (2015). New psychoactive substances: catalysing a shift in forensic science practice? Philos. Trans. R. Soc. Lond B Biol. Sci. 370. 10.1098/rstb.2014.0265 26101290 PMC4581009

[B35] Tettey J. N. A. Crean C. Ifeagwu S. C. Raithelhuber M. (2018). Emergence, diversity, and control of new psychoactive substances: a global perspective. Handb. Exp. Pharmacol. 252, 51–67. 10.1007/164_2018_127 29896655

[B36] Towers E. B. Hsu K. A. Qillawala E. I. Fraser S. D. Lynch W. J. (2024). Sex differences in the development of an opioid addiction-like phenotype: a focus on the telescoping effect. Biol. Psychiatry Glob. Open Sci. 4, 100373. 10.1016/j.bpsgos.2024.100373 39309210 PMC11416664

[B37] Vandeputte M. M. Glatfelter G. C. Walther D. Layle N. K. St Germaine D. M. Ujvary I. (2024). Characterization of novel nitazene recreational drugs: insights into their risk potential from *in vitro* micro-opioid receptor assays and *in vivo* behavioral studies in mice. Pharmacol. Res. 210, 107503. 10.1016/j.phrs.2024.107503 39521025 PMC11655282

[B38] Vevelstad M. Krabseth H. M. Haneborg A. M. Gundersen P. O. M. Frost J. Oiestad A. M. L. (2026). Nitazene-related deaths in Norway 2021-2024. Forensic Sci. Int. 380, 112794. 10.1016/j.forsciint.2025.112794 41477999

[B39] Walter L. A. Bunnell S. Wiesendanger K. Mcgregor A. J. (2022). Sex, gender, and the opioid epidemic: crucial implications for acute care. AEM Educ. Train. 6, S64–S70. 10.1002/aet2.10756 35783078 PMC9222889

[B40] Wei A. D. Burgraff N. J. Oliveira L. M. Moreira T. S. Ramirez J. M. (2026). Fentanyl blockade of K(+) channels contributes to wooden chest syndrome. J. Physiol. 604, 582–604. 10.1113/JP289107 41110851 PMC13078850

[B41] Wickham R. J. (2017). Cancer pain management: opioid analgesics, part 2. J. Adv. Pract. Oncol. 8, 588–607. 10.6004/jadpro.2017.8.6.3 30310721 PMC6167083

[B42] Wightman R. S. Perrone J. Scagos R. Hallowell B. D. Krieger M. Li Y. (2021). Toxicological and pharmacologic sex differences in unintentional or undetermined opioid overdose death. Drug Alcohol Depend. 227, 108994. 10.1016/j.drugalcdep.2021.108994 34482038 PMC8464519

[B43] Zagorski C. M. Myslinski J. M. Hill L. G. (2020). Isotonitazene as a contaminant of concern in the illegal opioid supply: a practical synthesis and cost perspective. Int. J. Drug Policy 86, 102939. 10.1016/j.drugpo.2020.102939 32977186

