# Supplementary figures and images for: Acute toxicity and sex-dependent lethality of isotonitazene in fischer 344 rats
Source: Front Toxicol. 2026 Apr 1;8:1818684. doi: 10.3389/ftox.2026.1818684 (PMC13078734; doi:10.3389/ftox.2026.1818684)

## Slide 1
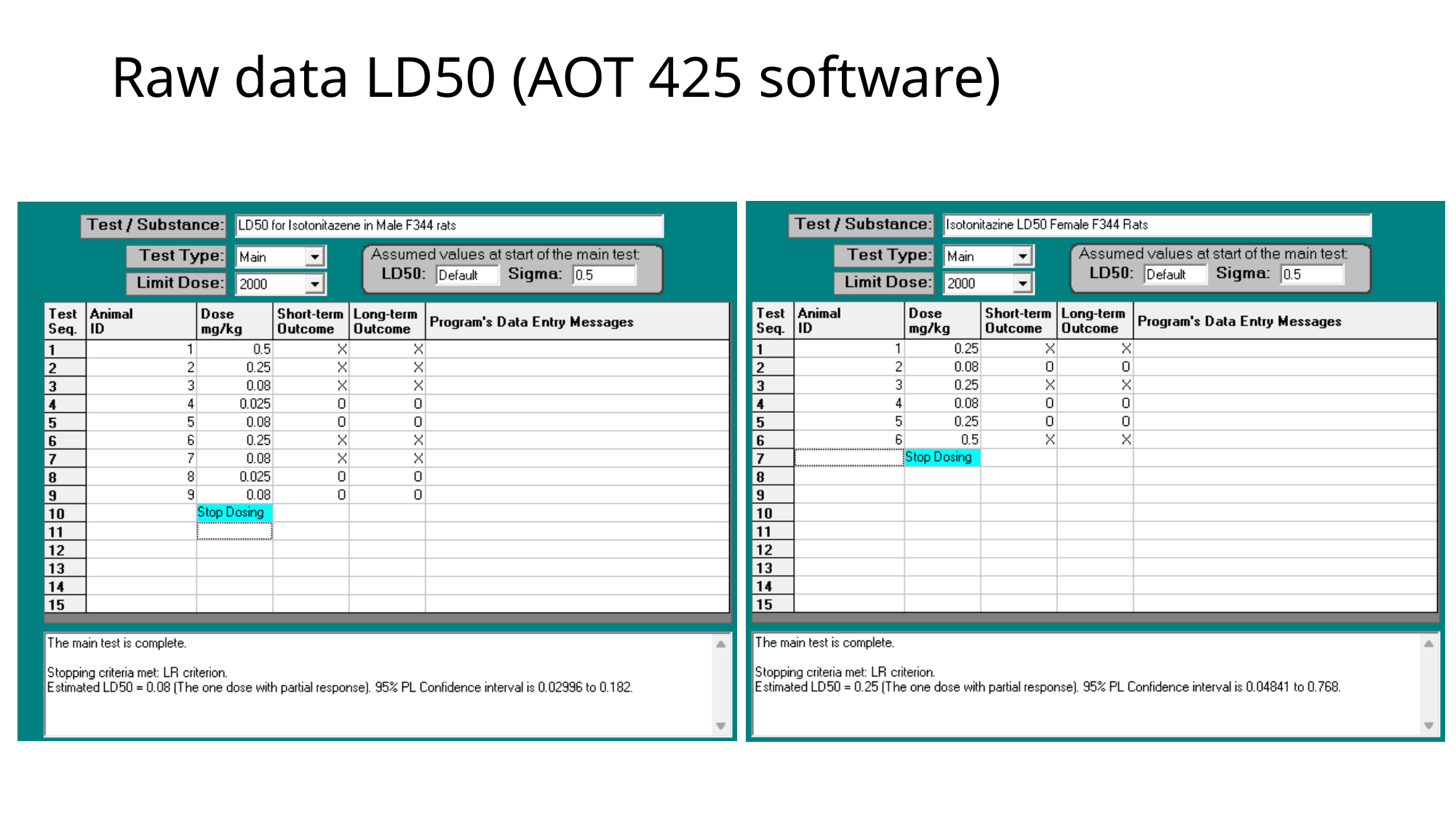

# Raw data LD50 (AOT 425 software)

## Slide 2
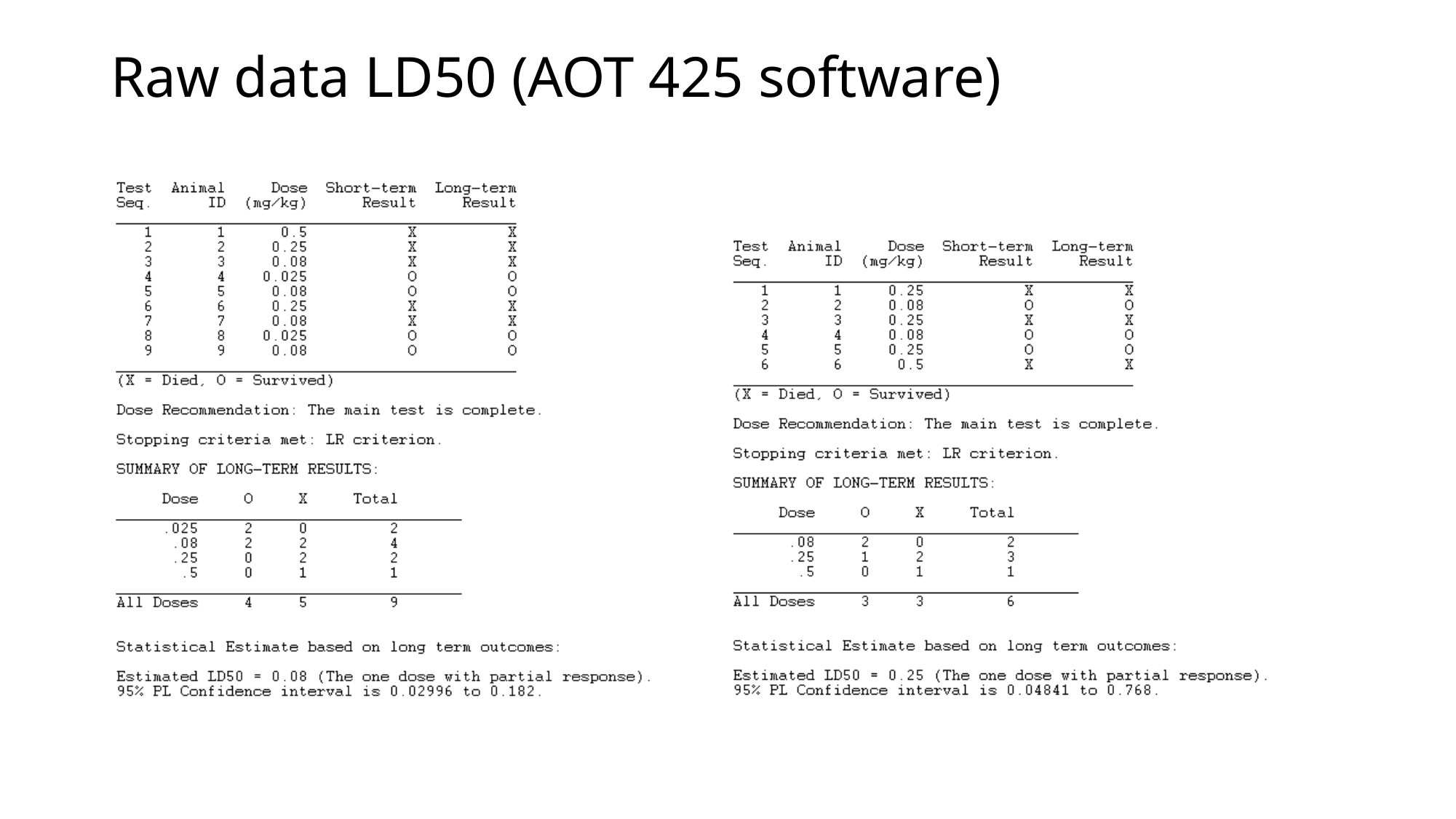

# Raw data LD50 (AOT 425 software)

Supplement: Supplementary file 1 [file Presentation1.pptx]
